# Supplementary material for: Screening of Candidate Housekeeping Genes in Uterus Caruncle by RNA-Sequence and qPCR Analyses in Different Stages of Goat (Capra hircus)
Source: Animals (Basel). 2023 Jun 6;13(12):1897. doi: 10.3390/ani13121897 (PMC10295728; doi:10.3390/ani13121897)
Supplement: Supplementary file 1 [file animals-13-01897-s001.zip › animals-2292553-supplementary/animals-2292553-supplementary/supplementary materials animals-2292553/animals-2292553-figures.pdf]

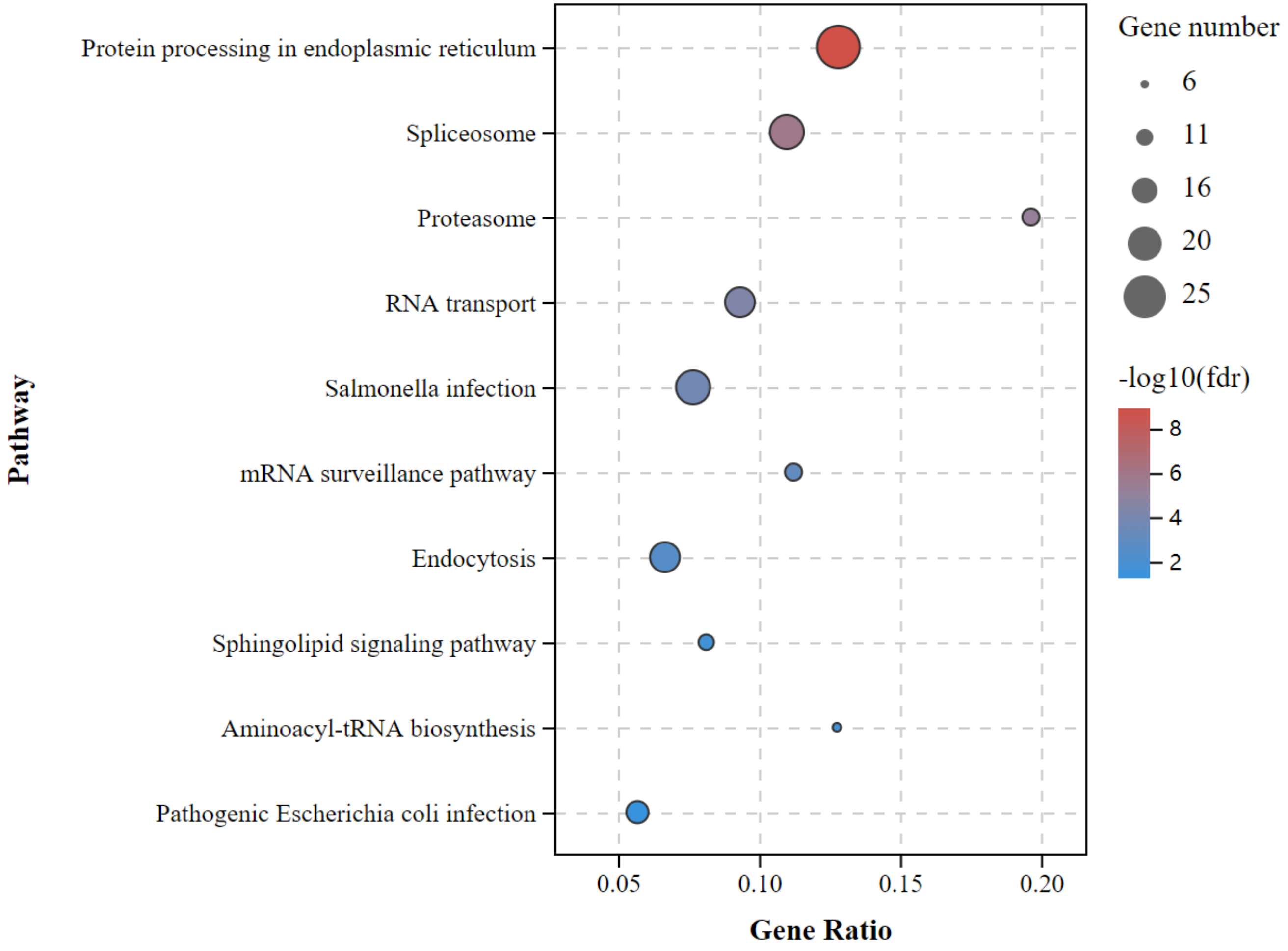


**Figure S1**. KEGG (Kyoto Encyclopedia of Genes and Genomes) enrichment analysis of HKGs.

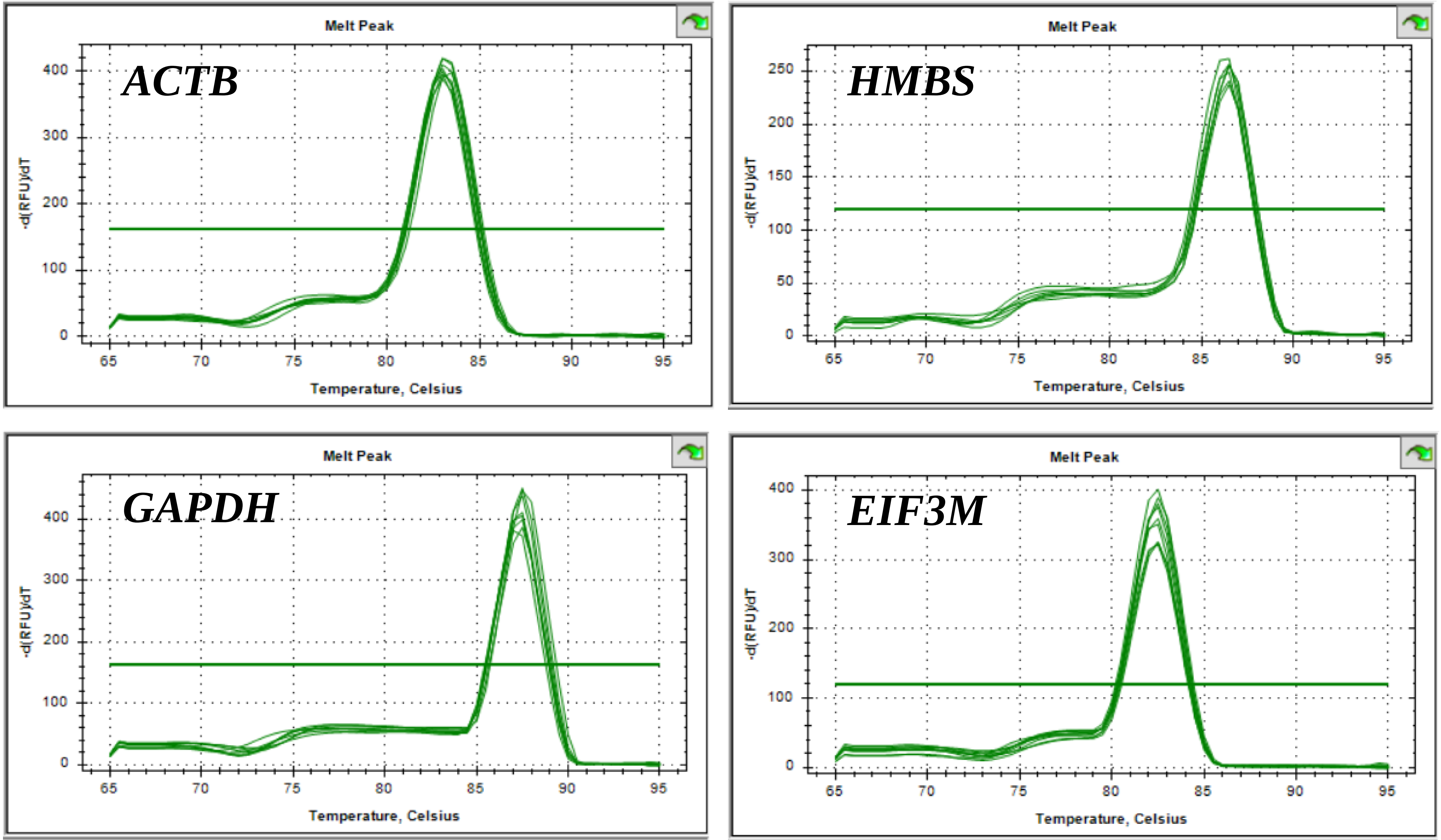

Melt Peak
ACTB
-d(RFU)/dT
400
300
200
100
0
65
70
75
80
85
90
95
Temperature, Celsius
Melt Peak
HMBS
-d(RFU)/dT
250
200
150
100
50
0
65
70
75
80
85
90
95
Temperature, Celsius
Melt Peak
GAPDH
-d(RFU)/dT
400
300
200
100
0
65
70
75
80
85
90
95
Temperature, Celsius
Melt Peak
EIF3M
-d(RFU)/dT
400
300
200
100
0
65
70
75
80
85
90
95
Temperature, Celsius

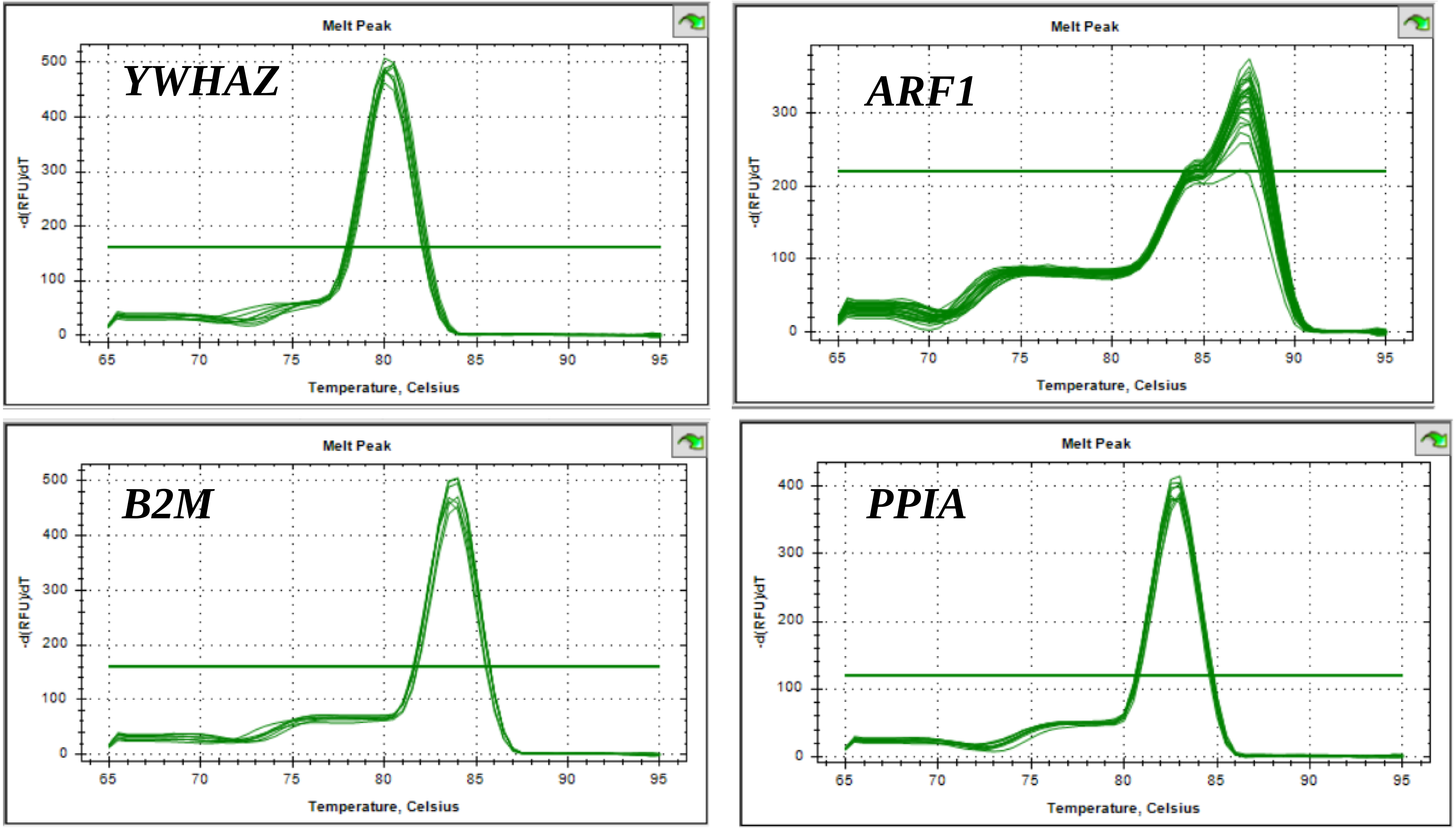

Melt Peak
YWHAZ
-d(RFU)/dT
500
400
300
200
100
0
65
70
75
80
85
90
95
Temperature, Celsius
Melt Peak
ARF1
-d(RFU)/dT
300
200
100
0
65
70
75
80
85
90
95
Temperature, Celsius
Melt Peak
B2M
-d(RFU)/dT
500
400
300
200
100
0
65
70
75
80
85
90
95
Temperature, Celsius
Melt Peak
PPIA
-d(RFU)/dT
400
300
200
100
0
65
70
75
80
85
90
95
Temperature, Celsius

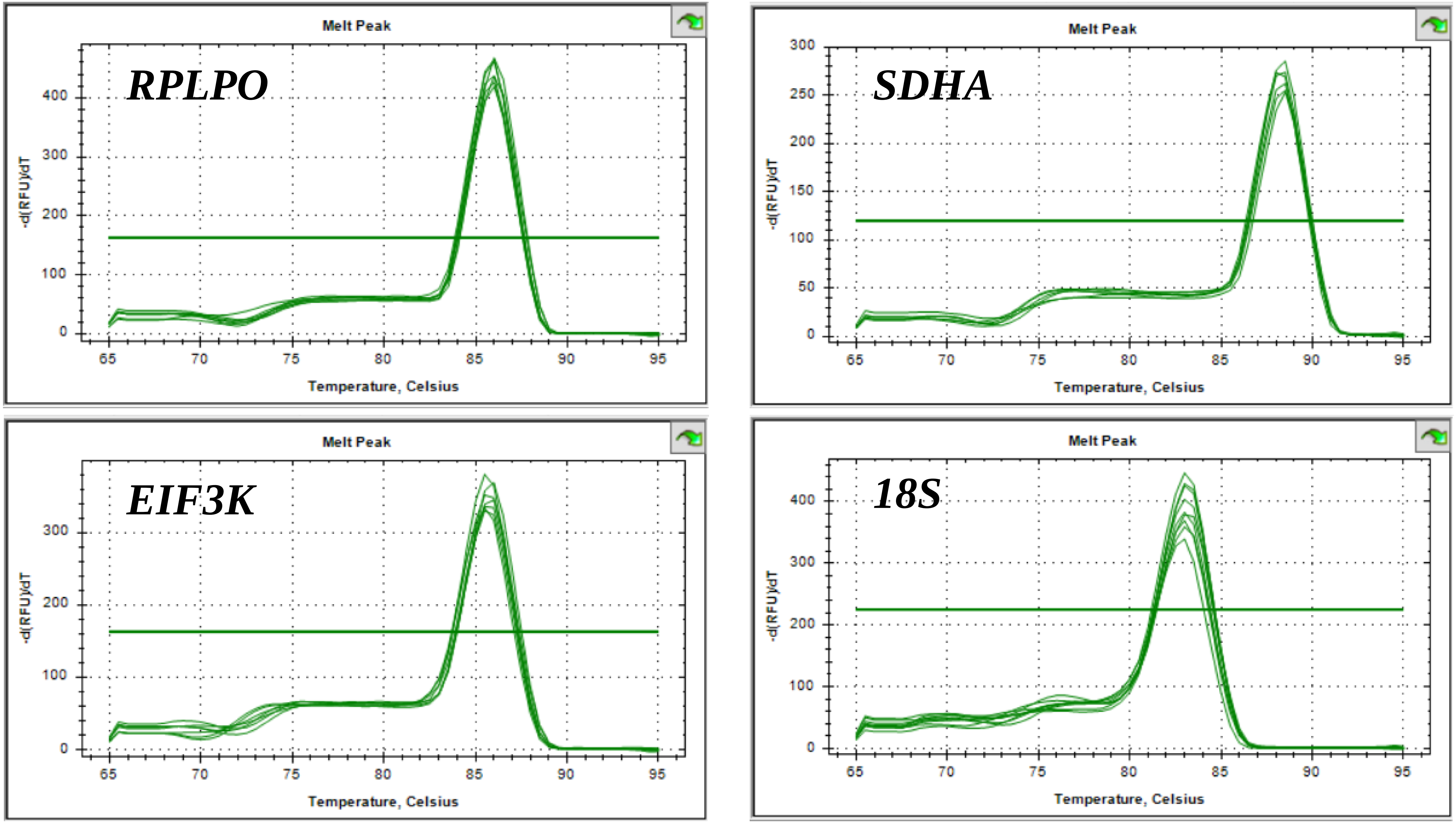

Melt Peak
RPLPO
-d(RFU)/dT
Temperature, Celsius
Melt Peak
SDHA
-d(RFU)/dT
Temperature, Celsius
Melt Peak
EIF3K
-d(RFU)/dT
Temperature, Celsius
Melt Peak
18S
-d(RFU)/dT
Temperature, Celsius

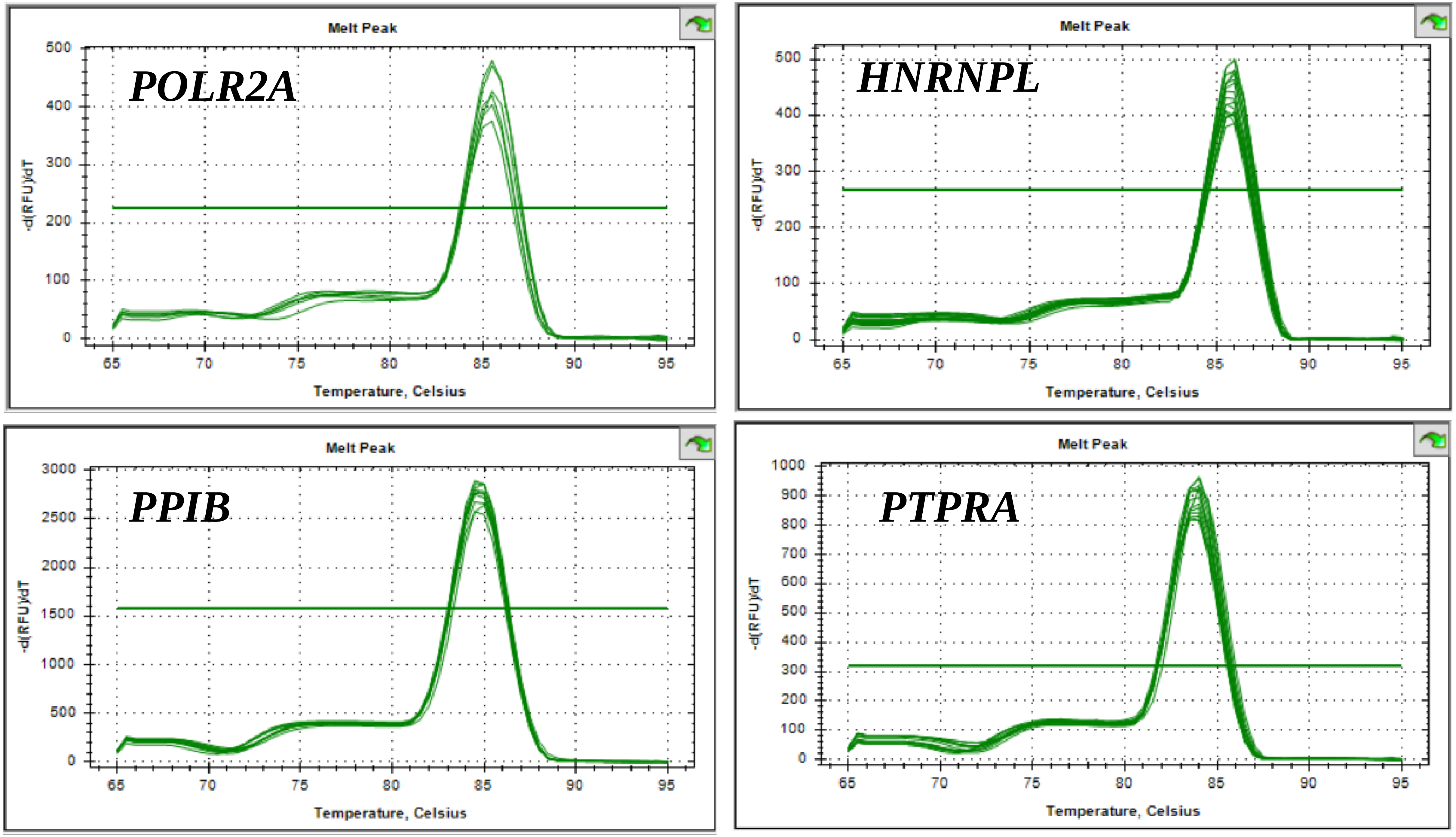

Melt Peak
POLR2A
-d(RFU)/dT
500
400
300
200
100
0
65
70
75
80
85
90
95
Temperature, Celsius
Melt Peak
HNRNPL
-d(RFU)/dT
500
400
300
200
100
0
65
70
75
80
85
90
95
Temperature, Celsius
Melt Peak
PPIB
-d(RFU)/dT
3000
2500
2000
1500
1000
500
0
65
70
75
80
85
90
95
Temperature, Celsius
Melt Peak
PTPRA
-d(RFU)/dT
1000
900
800
700
600
500
400
300
200
100
0
65
70
75
80
85
90
95
Temperature, Celsius

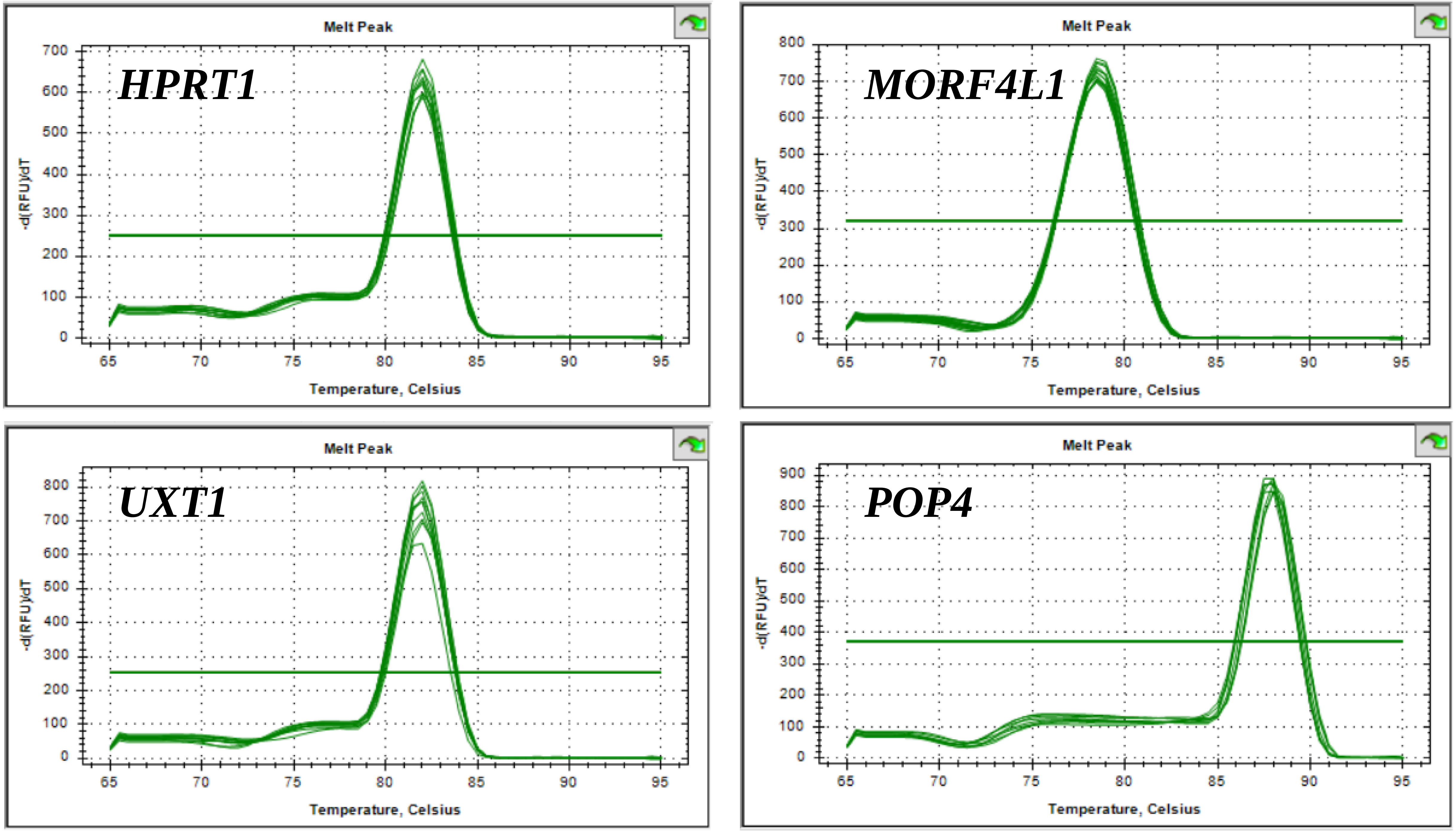

Melt Peak
HPRT1
-d(RFU)/dT
Temperature, Celsius
Melt Peak
MORF4L1
-d(RFU)/dT
Temperature, Celsius
Melt Peak
UXT1
-d(RFU)/dT
Temperature, Celsius
Melt Peak
POP4
-d(RFU)/dT
Temperature, Celsius

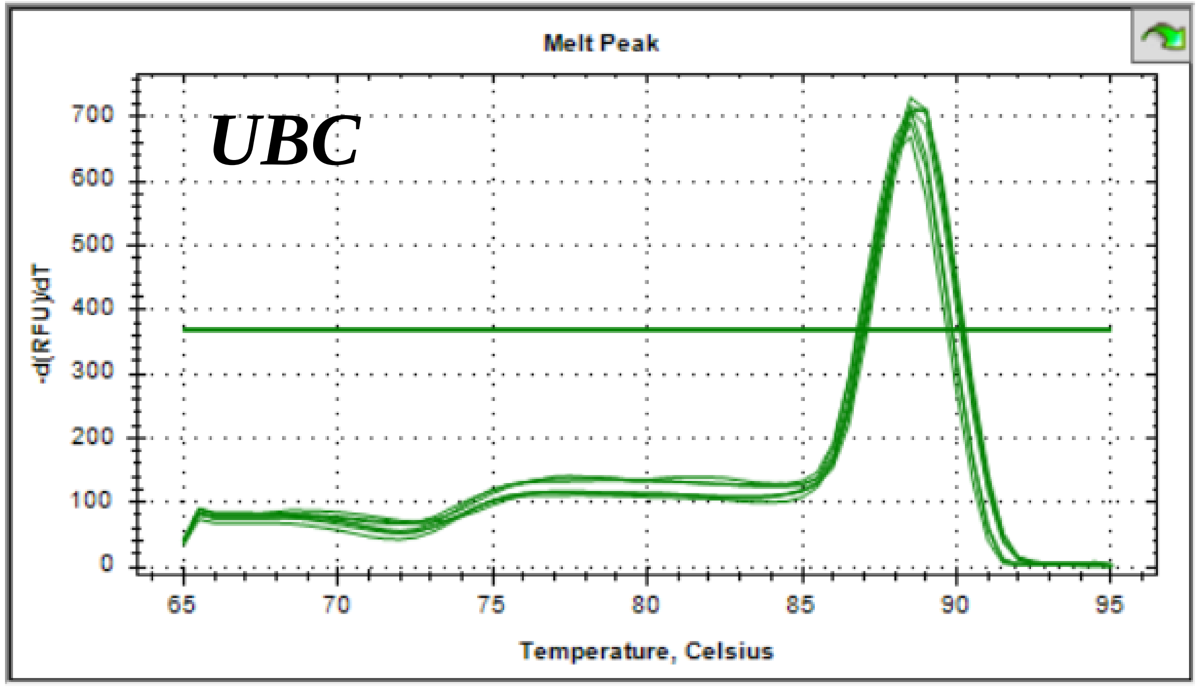

Melt Peak
UBC
-d(RFU)/dT
700
600
500
400
300
200
100
0
65
70
75
80
85
90
95
Temperature, Celsius


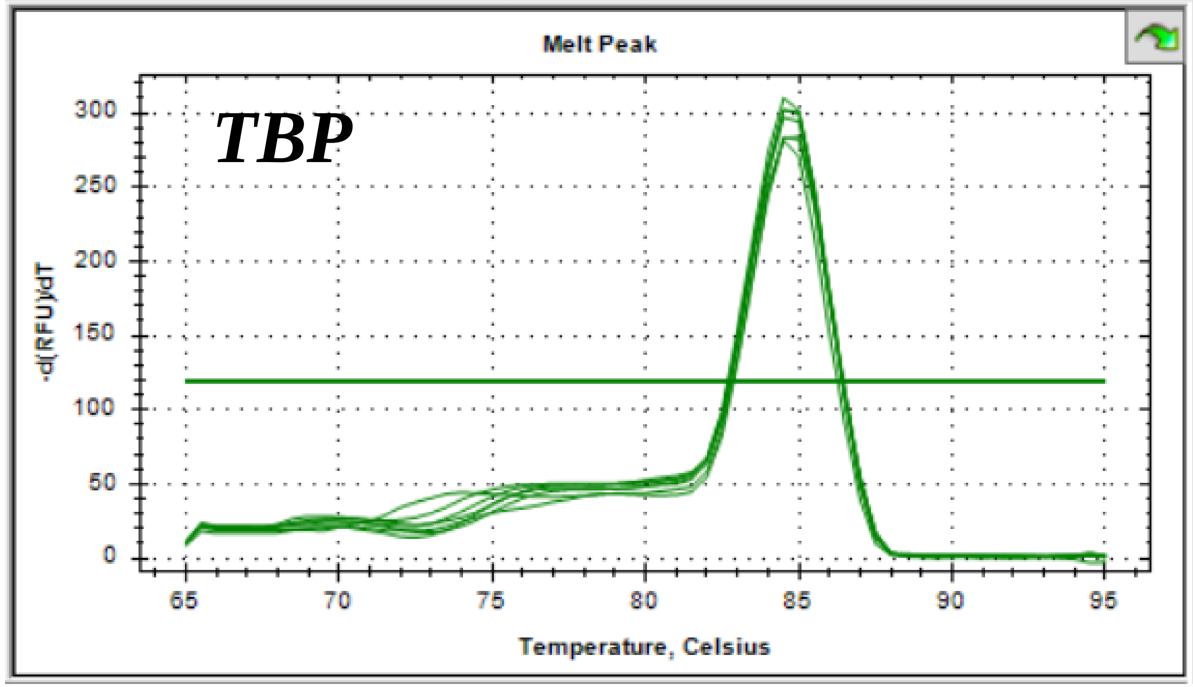

Melt Peak
TBP
-d(RFU)/dT
300
250
200
150
100
50
0
65
70
75
80
85
90
95
Temperature, Celsius

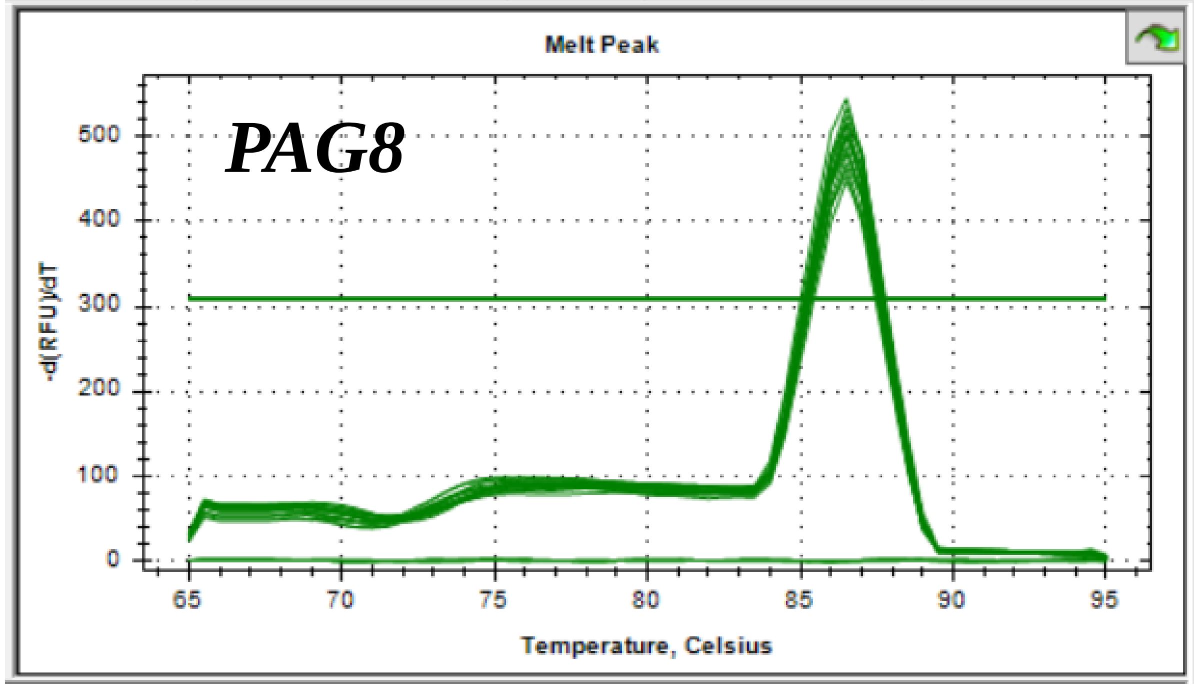


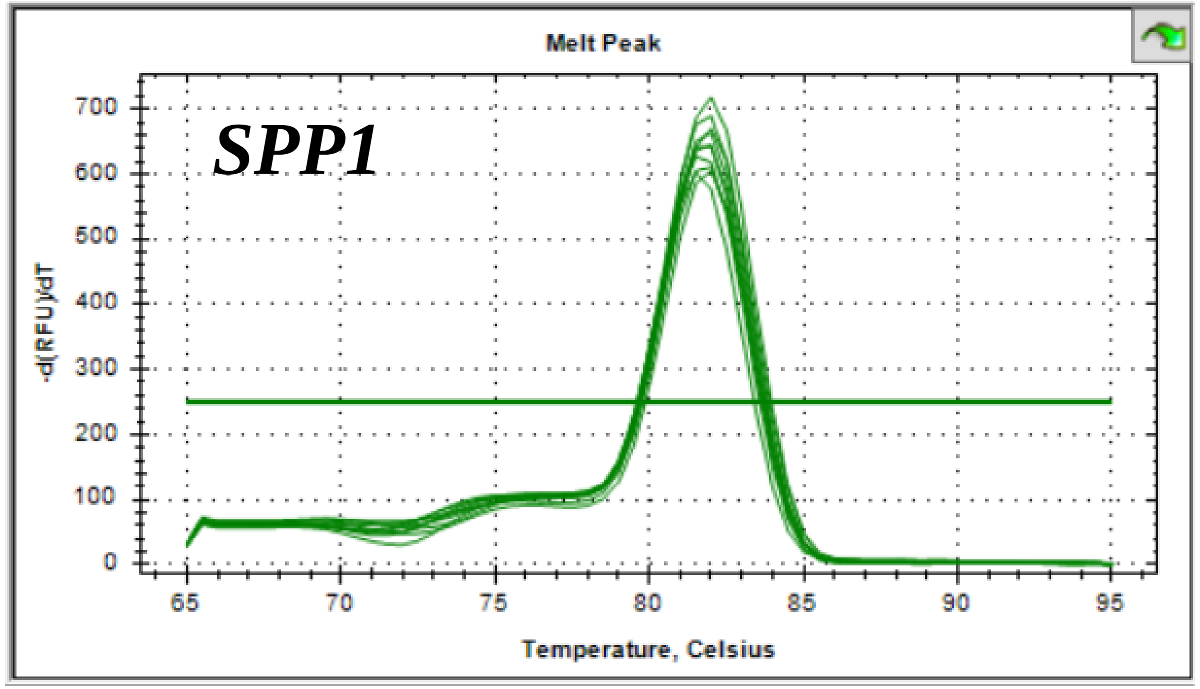


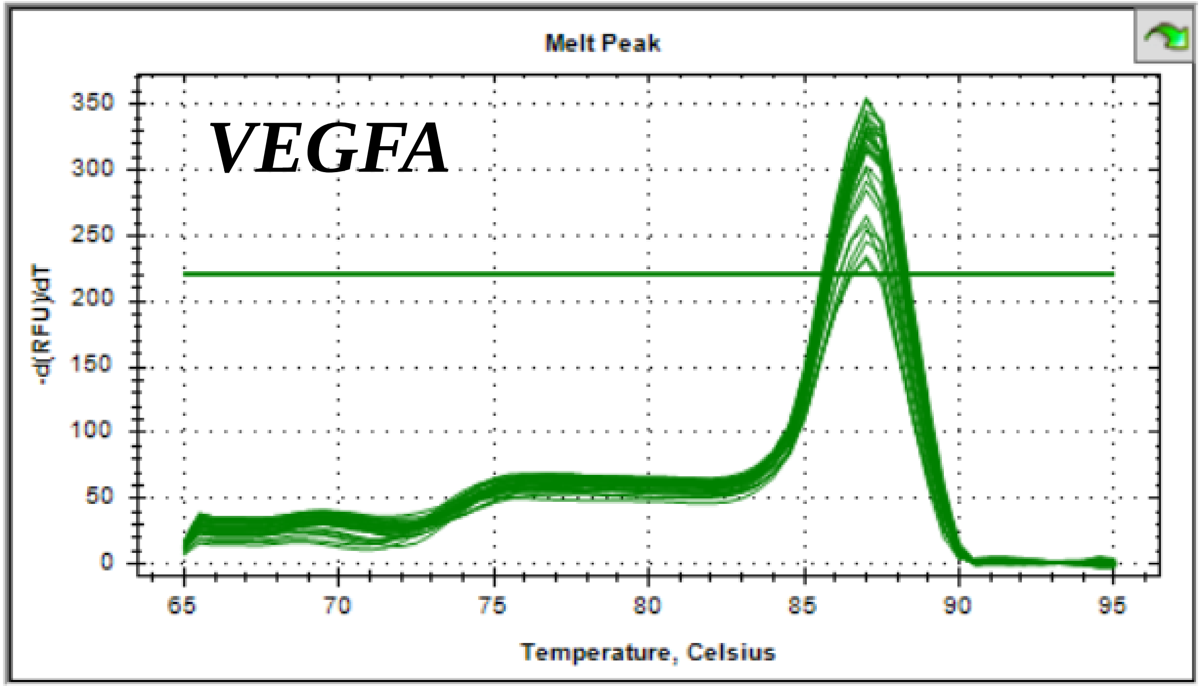


**Figure S2.** qRT-PCR melting curves of 22 HKGs and 3 target genes.
