# Supplementary material for: Screening of Candidate Housekeeping Genes in Uterus Caruncle by RNA-Sequence and qPCR Analyses in Different Stages of Goat (Capra hircus)
Source: Animals (Basel). 2023 Jun 6;13(12):1897. doi: 10.3390/ani13121897 (PMC10295728; doi:10.3390/ani13121897)
Supplement: Supplementary file 1 [file animals-13-01897-s001.zip › animals-2292553-supplementary/animals-2292553-supplementary/supplementary materials animals-2292553/Supplementary tables.pdf]

Table S1. Primer sequences information used in this study

Candidate Reference Gene name	NCBI gene ID	Forward primer (Sequence 5'–3')	Reverse primer (Sequence 5'–3')	Tm (℃)	Production size (bp)	Efficiency (%)
ACTB	XM_018039831.1	CCTGCGGCATTCACGAAACTAC	ACAGCACCGTGTTGGCGTAGAG	61.3	87	97.4
GAPDH	XM_005680968.3	GCAAGTTCCACGGCACAG	GGTTCACGCCCATCACAA	61.3	249	95.7
18S RNA	DQ149973	TAATCCCGCCGAACCCCATT	GGTGTGTACAAAGGGCAGG	61.3	125	93.3
YWHAZ	XM_018058314.1	AGACGGAAGGTGCTGAGAAA	CGTTGGGGATCAAGAACTTT	57.0	123	101.9
POLR2A	NM_001206313.1	AGAGGTGGTGGACAAGATGG	ACACCTTGCTGATCTGCTCT	60.0	104	94.8
B2M	DQ386890	TGTCCCACGCTGAGTTCACT	TGAGGCATCGTCAGACCTTGA	60.0	137	103.7
HMBS	AB232537	GCGGGAGAGCCCCTATGA	AGCTGCGGCCAGGATGAT	60.0	229	98.9
EIF3M	XM_018059285.1	CTGTGCGAGAAACTGGTCAA	ATATACTGGATGGCCCCACA	60.0	164	95.7
POP4	XM_005692232.3	TTCTGGCACAAGGAACGTCA	CATGGTAGGCCACACTCGTC	60.0	165	102.5
HPRT1	XM_013976270.1	CACTGGGAAGACAATGCAGA	ACACTTCGAGGGGTCCTTTT	60.0	102	99.5
TBP	XM_018053502.1	TCGCCAAGAATAGTGTGCTG	CCGTAAGGCATCATTGGACT	61.3	202	95.7
UBC	XM_005691342.3	TGCTTTTCTCTTTAGAGGGTGGGA	TCTGCATTGTCAATTGGTGTTGG	61.0	201	103.2
PPIA	XM_018047035.1	AAGTCCCGAAGACAGCAGAA	GATGCCAGGACCTGTATGCT	60.0	209	98.8
SDHA	XM_018065656.1	AGACGTTCGACAGGGGAATG	CTGGACGGGCTTGGAGTAAT	60.0	185	97.5
RPLP0	NM_001012682	CAACCCTGAAGTGCTTGACAT	AGGCAGATGGATCAGCCA	60.0	227	96.1
EIF3K	NM_001034489	CCAGGCCCACCAAGAAGAA	TTATACCTTCCAGGAGGTCCATGT	60.0	125	99.1
PPIB	XM_005685667	ACACCAACGGCTCCCAGT	AGGCTTGTCCCGACCATC	60.0	143	104.5
SPP1	XM_013964504.2	TTCTGGCAGCTCTGAGGAAA	TGCTTCTGAGATGGGTCAGG	59.1	87	102.5
PAG-8	NM_001285722.1	TCCCACGGATGTCAGGCCA	TAGTCAGGGGGCACAGG	60.2	200	99.7
VEGFA	NM_001287035.1	GCAAGAAAATCCCTGTGGGC	TGGGTCGTTCTGTGTCAGTC	59.7	243	102.2
ARF1	D87898.1	GACCTCCCCAATGCCATGAA	ATGTACCAGTTCCTGTGGCG	60.5	80	100.9
MORF4L1	NM_001357780.1	GTGCTTTCATGGGCCTCTTC	ACCCATTCATCCCAATTTTTATTCC	60.4	118	98.3
HNRNPL	NM_001402936.1	CTGATTGACGGTGTGGTGGA	CGGGAGATTTTCTGGCTGGT	60.8	218	99.7

Table S2. The pairwise variation in 17 HKGs for the determination of the number of HKGs

V_n/V_{n+1}	Stage1	Stage2	Stage3	Total
V2/3	0.008	0.023	0.013	0.020
V3/4	0.005	0.020	0.010	0.014
V4/5	0.003	0.016	0.008	0.013
V5/6	0.004	0.013	0.007	0.012
V6/7	0.004	0.011	0.007	0.011
V7/8	0.003	0.009	0.007	0.009
V8/9	0.004	0.010	0.006	0.009
V9/10	0.003	0.010	0.006	0.008
V10/11	0.003	0.010	0.005	0.008
V11/12	0.003	0.009	0.005	0.007
V12/13	0.003	0.010	0.005	0.007
V13/14	0.004	0.009	0.005	0.008
V14/15	0.004	0.008	0.006	0.007
V15/16	0.004	0.008	0.006	0.009
V16/17	0.005	0.010	0.012	0.010
